# Dynamic graph-based quantum feature selection for accurate fetal plane classification in ultrasound imaging

**DOI:** 10.1038/s41598-025-26835-y

**Published:** 2025-11-22

**Authors:** S. Priyadharshni, V. Ravi

**Affiliations:** 1 School of Electronics Engineering, Vellore Institute of Technology, Chennai, 600127 India; 2 Centre for Neuroinformatics, School of Electronics Engineering., Vellore Institute of Technology , Chennai, 600127 India

**Keywords:** Fetal ultrasound classification, Pre-trained deep learning model, Quantum entanglement, Dynamic graph feature selection, Pre-natal diagnosis, Computational biology and bioinformatics, Engineering, Health care, Mathematics and computing, Medical research

## Abstract

Accurate classification of fetal biometric planes in ultrasound imaging is more important for effective prenatal screening and early diagnosis of fetal abnormalities. To enhance the diagnostic efficiency, the research proposed a novel method called “Dynamic Graph-Based Quantum Feature Selection” (DG-QFS) framework to improve the classification performance by integrating the quantum computing principles. Features are extracted from ultrasound images using a pre-trained deep learning model and processed through a quantum-driven feature selection pipeline that models the inter-feature relationships using dynamically entangled multi-qubit graphs. In the DG-QFS method, qubits represent extracted deep feature nodes, while a quantum entanglement score-based dynamic graph captures the complex dependencies. Entanglement score and dynamic graph centrality are used to select the most informative features. The refined feature set is classified using a lightweight multi-layer perceptron (MLP), stochastic gradient descent with adaptive learning rate. Examined the proposed model on a fetal plane ultrasound scan dataset, including 12,400 images of six categorical planes, such as brain, thorax, abdomen, femur, maternal cervix, and other views. Experimental results demonstrate that the proposed model achieves a classification accuracy of 96.73%, significantly outperforming baseline deep learning and conventional feature selection techniques regarding accuracy, generalization, and interpretability.

## Introduction

 Accurate classification of fetal anatomical planes in ultrasound scans (US) is important in antenatal diagnosis. Standard fetal biometric planes of six classes, such as the brain, thorax, abdomen, femur, maternal cervix, and the other category, provide essential measurements for monitoring fetal growth, detecting congenital anomalies, and assessing gestational age. However, manual interpretation of US is often prone to human false detection, inter-observer variability, and time-consuming problems. These challenges represent the need for automatic intelligent systems to assist accurate medical decision-making. Deep learning models have emerged as a highly powerful tool in medical image analysis in many applications^1–3^. Lightweight models have shown remarkable performance in extracting relevant features from ultrasound images, owing to their computational efficiency and architectural simplicity. Nevertheless, deep models often generate redundant or noisy features, which may degrade classification performance and limit model interpretability.

Feature selection is a key process that aims to select the most relevant features from the data while eliminating the redundant ones and improving the fetal plane classification accuracy, model robustness, and solving the combinatorial problems. Some conventional feature selection methods, such as principal component analysis (PCA), filter methods, wrapper techniques, and entropy-based measures, are limited in capturing complex, non-linear relationships among features^4–6^. Quantum computing applies a new approach to solving this issue by utilizing quantum properties like superposition, entanglement, and quantum coherence. In particular, multi-qubit entanglement is used to have interdependencies between features in high-dimensional data. Quantum-driven feature selection methods have shown promise in enhancing the performance of machine learning models by representing correlations more effectively than classical methods. Therefore, the proposed model is designed with modularity in mind, making it suitable for deployment in clinical settings in case of ultrasound-specific noise disturbances encountered commonly during prenatal scans, and serves as a decision-support tool for automated fetal plane identification in practical obstetric workflows. Based on the limitations observed in conventional and recent deep-learning-based ultrasound frameworks, the following key contributions distinctly highlight the innovations introduced in the proposed DG-QFS approach.

The Key Contributions of the Study,

1. A novel framework named Dynamic Graph-based Quantum Feature Selection (DG-QFS) is proposed to improve the classification of Fetal US planes, which merges the quantum entanglement theory with a graph-based model to identify the most important features for accurate classification. The model with feature extraction using a deep learning model as a backbone is more efficient for medical imaging tasks.
2. The obtained relevant features are mapped into a graph architecture, where each node signifies a feature, and edges are weighted based on quantum entanglement score and centrality. The graph-node structure obtains both local and global relationships between features and provides a dynamic selection process to identify the most significant features. It analyzes node connectivity, centrality, and interaction strength.
3. The selected features are classified by a multi-layer perceptron (MLP) classifier configured with an adaptive learning rate, stochastic gradient descent, and ensuring stable convergence. The hybrid framework provides quantum-enhanced feature selection with a deep learning backbone to deliver high performance.
4. The Experimental results confirm that the DG-QFS model achieves a classification accuracy of.

96.73%, calculated with other metrics such as precision, recall, and F1 score, and feature selection metrics, significantly outperforming baseline deep learning models and existing feature selection methods in terms of accuracy, computational complexity, and generalization.

5. The strong inter-feature relationships through quantum entanglement correlation and updated graph weights, the proposed work improves interpretability, which is an essential factor in medical AI applications, where clinical explainability is a challenging case in fetal plane classification. The subsequent sections of the paper are organized as follows: Sect. 2 gives a survey of fetal plane classification with deep learning techniques, integrated quantum-classical CNN models, and feature selection models, and highlights the limitations of existing models. Section 3 provides information about the proposed DG-QFS model’s structure, with its diagram. Section 5 shows the experimental results and discussions about the proposed model through graphs and tables. Section 6 concludes and explains future work.

## Related works

The precise classification of fetal biometric planes in ultrasound imaging is critical for ensuring early and effective prenatal diagnostics. Over the past five years, many deep learning techniques have been proposed to automate this classification task and significantly improve the diagnostic efficiency of fetal biometric planes. Traditional convolutional neural networks (CNNs) are widely adopted due to their strong spatial-feature learning capabilities. Krishna and Kohil have explored a stacked ensemble approach using deep learning techniques for the accurate identification of fetal planes using a multiclass US dataset and resulting in good performance in the classification of fetal planes^7^. Rahman et al. constructed a neural network developed by the Dempster-Shafer theory and adopting fuzzy-based contrast enhancement, combined with explainable AI techniques, which results in better classification in improved quality of images^8^.

Recent developments are explored in Transformer techniques for better feature representation in the US data. Sarker et al. proposed transformer-driven cross-covariance attention mechanisms for the effective classification of maternal-fetal and brain classes^9^. Burgos-Artizzu et al. assessed the performance of deep CNNs for the automatic identification of standard fetal US planes, paving the way for CNN-driven automation in this domain^10^. The few research works mainly focused on feature selection and optimization techniques for further development in classifying the biometric planes. Rathika et al. assessed an optimized feature selection strategy to refine neural network inputs for fetal plane classification and thereby reduce the overfitting^11^. Rauf et al. developed a residual network consisting of 82 layers deep, combined with an optimization algorithm for effective classification of three classes of brain and six common fetal planes^12^. Al-Razgan et al. explained the advantage of attention based convolution to maximize the spatial understanding of fetal structures, showcasing improvements in both performance and interpretability^13^. Advanced research in fetal US image analysis has made significant progress through deep learning and quantum-enhanced techniques.

Convolutional neural networks are widely used for standard plane classification^14,15^, anatomical structure detection^16^, and biometric estimation^17^. Enhancements such as attention mechanisms^18^ and multi-task learning^19^ have improved model precision and generalization. Generative models like GANs have supported fetal brain image synthesis using anatomical priors^20,21^, and datasets from^22^ supported standardized model training^23–26^.Explaining the hybrid models in capturing enriched features for better classification by an integrated approach called the Quantum convolutional.

neural network. To focus on the challenges of architectural complexity and strong correlation across features^27^, the graph-based approach^28^ and, for better visualization, AI explainability methods^29^ are explored.

Feature selection is a pivotal step in machine learning algorithms, aiming to reduce dimensionality, enhance model interpretability, and improve classification accuracy in noisy scenarios. Recent years have witnessed benchmark progress in combining quantum-inspired methods for optimization and graph-based approaches for robust and efficient feature selection across diverse applications, from medical imaging to several other applications. Quantum computing principles have inspired several novel optimization algorithms for feature selection, possessing quantum phenomena such as superposition, entanglement, and quantum parallelism. Zhong et al. Elaborate on the adaptive feature selection with an artificial bee colony technique based on a quantum-driven equilibrium optimizer, which results in optimal features for better classification and solves the low convergence problem. This method is tested with 25 datasets, including medical image datasets, and compared with several metaheuristic algorithms and with different equilibrium optimizers^4^. Mandal et al. designed an approach for selecting an enhanced feature subset for high classification performance. It employs a Quantum-assisted Owl search algorithm to select an optimal feature selection, which was tested with twelve public datasets and compared the measurements with classical optimization techniques^30^.

Pu et al. presented a methodology that improves the quality of images by the integration of the quantum-assisted feature selection with a generative adversarial network. It is evaluated on the aerial images by testing the resolution using parameters such as t-distributed stochastic neighbor embedding, structural similarity index metric, and peak signal-to-noise ratio. The experimental outcomes show a significant improvement and better similarity index compared to conventional methods in a statistical way^31^. Abdul Hussien et al. introduce a Quantum-Inspired Genetic Algorithm for the application of legal identification to prevent fraud activities. The feature selection using quantum concepts shows good convergence compared to traditional genetic algorithms by balancing exploration and exploitation. Tested on numerous datasets and obtained a 10–20% improvement in equal error rate with low computational cost. Classical methods independently rank the features, show suboptimal classification performance, and lead to computational complexity for large datasets^32^. Li et al. Solving the integer programming problem by Quantum annealers along with a wrapper algorithm to optimize the Quadratic unconstrained binary Optimization (QUBO) parameters^33^.

Turati et al. focus on solving a quadratic feature selection problem using the efficient optimization algorithm known as, Quantum Approximate Optimization Algorithm (QAOA). The QUBO problem is aligned with a Hamiltonian, and it is optimized by QAOA to select the optimal features. It is measured on several datasets by using quantum simulators and IBM’s 7-qubit quantum computer, which demonstrates the feasibility of this approach. Future work can enhance performance by exploring diverse classifiers and better classical optimizers for QAOA^34^. Quantum computing improves the analysis of data through its superposition and entanglement principles. Chikhaoui et al. have displayed a hybrid method utilizing a non-negative matrix factorization incorporating quantum theory for feature reduction, and a quantum support vector machine is used for classification. It reduces the data complexity and achieves high accuracy. Tested on several datasets and shows that it outperforms the traditional models^35^. Chen et al. investigated a meta-learning with quantum neural networks and are followed by quantum optimization algorithms are resulting in adaptive quantum optimization in the feature learning process^36^. Graph-theoretic models provide complex relationships among features by capturing various interactions, dependencies, and more about structural information, which are more crucial for multi-label and high-dimensional datasets.

Akhavan et al. designed an efficient feature selection method for machine learning applications by utilizing a Graph-based feature selector combined with a class-oriented feature map. It uses a decision tree and Support vector machine for classification and evaluated across five datasets^37^. Hatami et al. experimented with a graph-integrated feature selection method with an ant colony optimization (ACO) for filtering the features in multiple labels^38^. Akhiat et al. displayed a Graph feature selection method to capture the informative features, which enhances the classifier accuracy^39^.Dalvand et al. demonstrated a semi-supervised graph-based feature selection algorithm using PageRank by exploiting graph centrality to prioritize informative features in partially labeled datasets^40^. Cheng et al. examined dynamic node mechanisms within graph-based feature selection, emphasizing structural adaptability to changing data distributions^41^. Zhong et al. designed the model by combining the graph embedding orthogonal decomposition with collaborative particle swarm optimization for synchronous feature selection, by achieving superior feature subset coherence^42^. Jiang et al. introduce a quantum-inspired graph neural network to initialize the parameters in QAOA circuits by bridging graph learning and quantum optimization for enhanced feature selection applications^43^. Li et al. implemented a graph-theoretic feature selection by the QAOA approach by capturing feature dependencies while harnessing quantum optimization speedups^44^. Yue et al. used a wrapper-based feature selection technique by combining quantum and swarm optimization with graph-based evaluation metrics by balancing exploration and exploitation in diverse datasets^5^.

Turaka and Panigrahy fused the chaotic adaptive particle swarm optimization with quantum-inspired genetic algorithms to obtain robust IoT intrusion detection feature selection by using hybrid quantum-classical metaheuristics for enhanced resilience to noisy data^45^. Shahriyar et al. further reinforced the hybrid paradigm, combining classical correlation metrics with quantum variational circuits. Multilabel learning performance is highly influenced by the input feature quality, which is degraded by irrelevant and redundant features. Most of the feature selection methods focus on removing the unwanted features and developing the feature interactions in case of data uncertainty^46^. Yin et al. Investigated a multilabel feature selection for a framework using fuzzy-based multi-neighborhood granules. It constructs a fuzzy set model with k-nearest neighbors to capture fuzzy dependencies based on features and their correlations. The mentioned approach analyzes the uncertainty between features and their labels in a model with multilabel data as a complete weighted graph^47^.

Nath et al. employed a quantum annealing process for stress detection and indicated the potential of quantum optimization in healthcare diagnostics^48^. Ye et al. presented a quantum self-organized feature mapping on a neural network for Grover’s search algorithm and blended with a quantum search with unsupervised feature learning^49^. He et al. employed the gradient-based optimization method for quantum architectural search, which optimized quantum circuit design for efficient feature selection^50^. Lu et al. proposed several transformer-based models for medical image diagnosis across different imaging modalities. The Lightweight Robust Alzheimer’s Disease Vision Transformer improves global feature extraction in brain magnetic resonance images by adaptively fusing tokens, prioritizing diagnostically relevant regions, and merging non-essential ones, achieving high accuracy for Alzheimer’s disease versus cognitively normal subjects and for cognitively normal versus mild cognitive impairment subjects, with reduced computational demand^51^. For chest X-ray classification, it integrates visual and textual information using report-guided multi-level alignment, large adaptive filters, and normalization to extract clinically meaningful features, achieving high accuracy while maintaining robustness and interpretability^52^. The Vision Transformer technique is used to address the overfitting issues in tuberculosis classification by discarding irrelevant tokens and stabilizing training with a randomized classifier, Outperforming state-of-the-art methods in efficiency and diagnostic performance^53^. Hekal et al. have shown recent advanced deep learning techniques performance in US image analysis.

For accurate breast tumor segmentation, employed a dual-decoder architecture with attention mechanisms^54^, while the another methodology is, the UNet model is integrated with Atrous Spatial Pyramid Pooling and Squeeze-and-Excitation blocks for fetal head segmentation application^55^. These techniques highlight the effectiveness of relevant feature extraction and attention mechanisms, which inspire feature learning in classification tasks.

The recent works [^56-73^] related to fetal plane classification show a trend towards automating and optimizing deep learning and quantum machine learning pipelines[^74‚75^] for practical use. The Deep learning solutions for fetal US applications face several challenges, including the scarcity of large annotated datasets and difficulty generalizing across different patients and devices. They are also sensitive to noise such as speckle and motion artifacts and it affects robustness. Recent Quantum technology has hardware constraints that limit the scalability of quantum feature selection methods in many applications. The interpretability of hybrid models in selecting relevant features needs further exploration to ensure transparent decision-making in sensitive domains such as medical diagnosis[^76^]. Integration with the conventional machine learning pipelines remains crucial for near-term applications.

Future research directions aim to develop an adaptive hybrid algorithm that balances quantum and classical resources dynamically and explores entanglement-based feature representations, and also increase the robustness in classification, and design application-specific quantum graph frameworks for real-world datasets.

## Proposed work

**Fig. 1:**
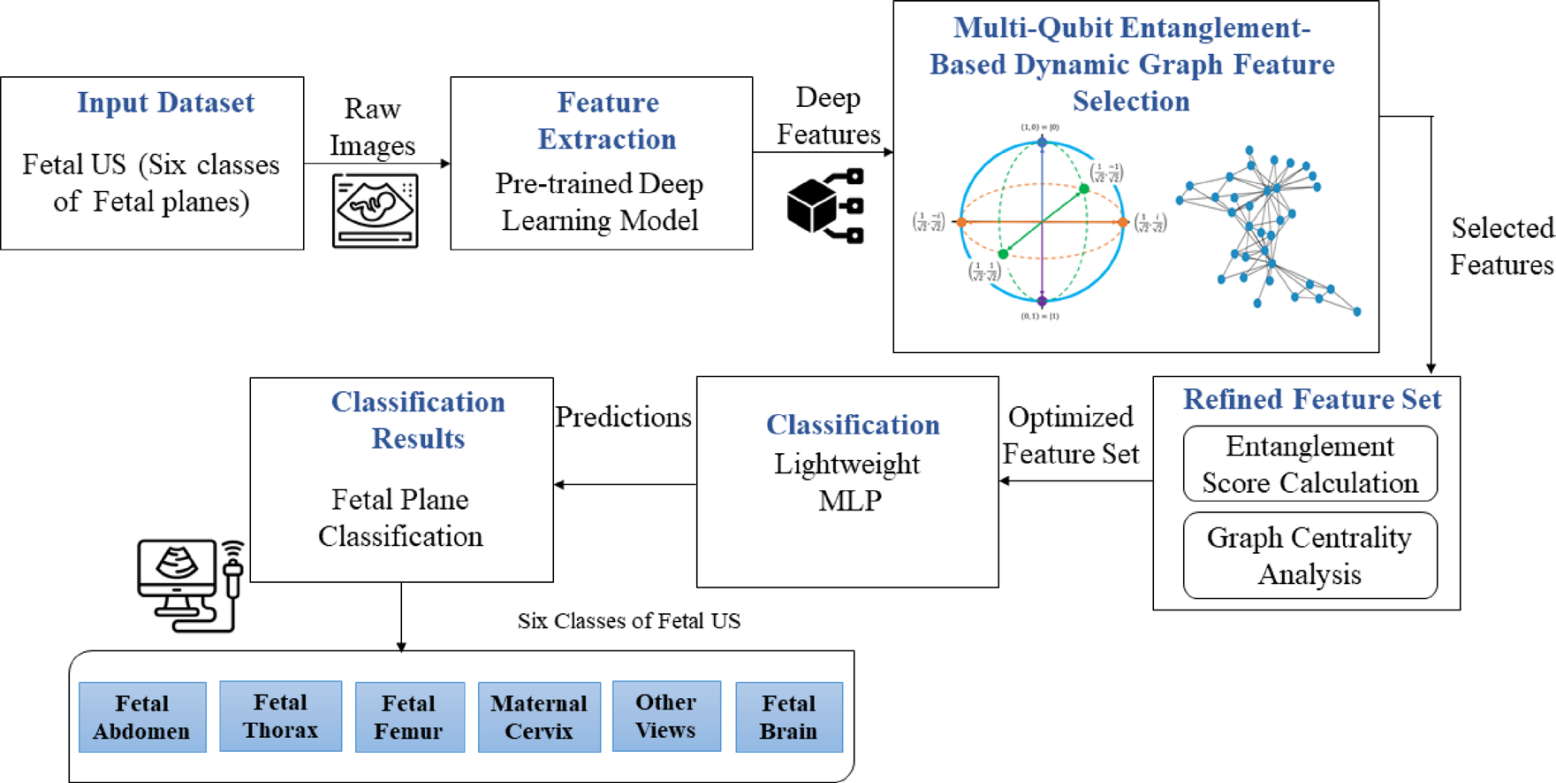
Overview of the proposed dynamic graph-based quantum feature selection (DG-QFS) framework for fetal plane US image classification. The framework integrates deep feature extraction with the quantum entanglement technique and dynamic graph-based feature selection to demonstrate that the relevant features are identified and refined through entanglement and graph centrality analysis, leading to improved classification accuracy and interpretability.

To address the challenge of accurately classifying fetal biometric planes in US images, a novel quantum-inspired feature selection framework called Dynamic Graph-based Quantum Feature Selection (DG-QFS). The proposed pipeline combines deep features extracted from a pre-trained deep learning model with a quantum entanglement-inspired graph modeling for efficient feature selection. Therefore, the refined feature set is obtained by quantum re-uploading the entanglement score and graph-theoretic metrics. The final feature set is classified using an Multi-Layer Perceptron (MLP) optimized with a stochastic adaptive learning strategy. The overall framework is outlined in the following sub-sections and shown in Fig. 1.

### Dataset description and preprocessing

The dataset used in the proposed (DG-QFS) model is fetal plane US images, which consist of 12,400 US images, categorized into six fetal plane classes, such as brain, thorax, abdomen, femur, maternal cervix, and miscellaneous views. It is publicly available and sourced from BCNatal, a maternal-fetal medicine center in Spain^10^. The ultrasound scans are acquired during the 18 to 40 weeks of gestation, and were obtained using six different machines equipped with curved transducers operating at frequencies between 3 and 7.5 MHz. All images, originally stored in DICOM format, are annotated by clinical experts and underwent a standardized preprocessing pipeline to ensure compatibility with deep learning and hybrid quantum-classical models. US images are resized to 224 × 224 × 3 pixels and normalized to [0, 1] intensity range by using min-max scaling for numerical stability. The dataset is employed with class weighting to handle class imbalance and is divided into 75% training and 25% testing sets.

**Table 1:** Details of fetal plane US images.

Category	No of images	No of patients	Medical use	No of training images	No of test images
Fetal abdomen	711	595	Fetal weight and growth.	533	178
Fetal brain	3092	1082	Brain development and size	2214	736
Fetal femur	1040	754	Fetal age and growth	780	260
Fetal thorax	1718	755	Thoracic circumference and lung development	1289	429
Maternal cervix	1626	917	Cervical length and predicting preterm birth risk	1219	406
Other	4213	734	Specific clinical needs.	3160	1053

**Fig. 2:**
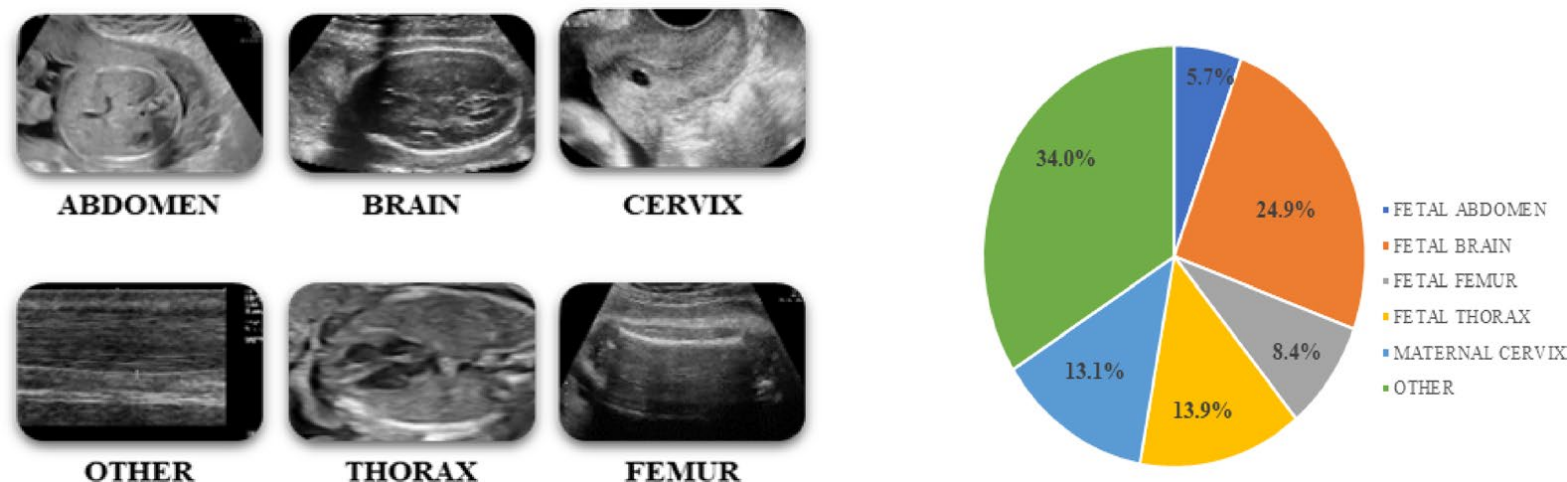
Sample images of Fetal plane US images and their category distribution. The pie chart depicts the distribution of US dataset in percentage by illustrating the largest representation from the “Other” and Brain classes.

The pie chart in Fig. 2 illustrates the distribution of fetal plane US images across six anatomical classes, with the largest portion (34%) categorized as “Other” views, followed by Fetal Brain images (24.9%) and Fetal Thorax (13.9%). The selective US images visually represent typical examples from each of these six classes, as shown in Fig. 2. US images with a blurriness score of less than 100 are considered in training or testing, and therefore, in the entire dataset, 143 (1.15%) US images are excluded. Table 1 depicts that the dataset comprises fetal plane US images categorized into six anatomical regions, each serving specific clinical purposes like assessing fetal growth, development, and maternal health. It has a split of training and test images, with a balanced distribution among patients and use cases for machine learning model development.

### Quantum-inspired framework

**Fig. 3:**
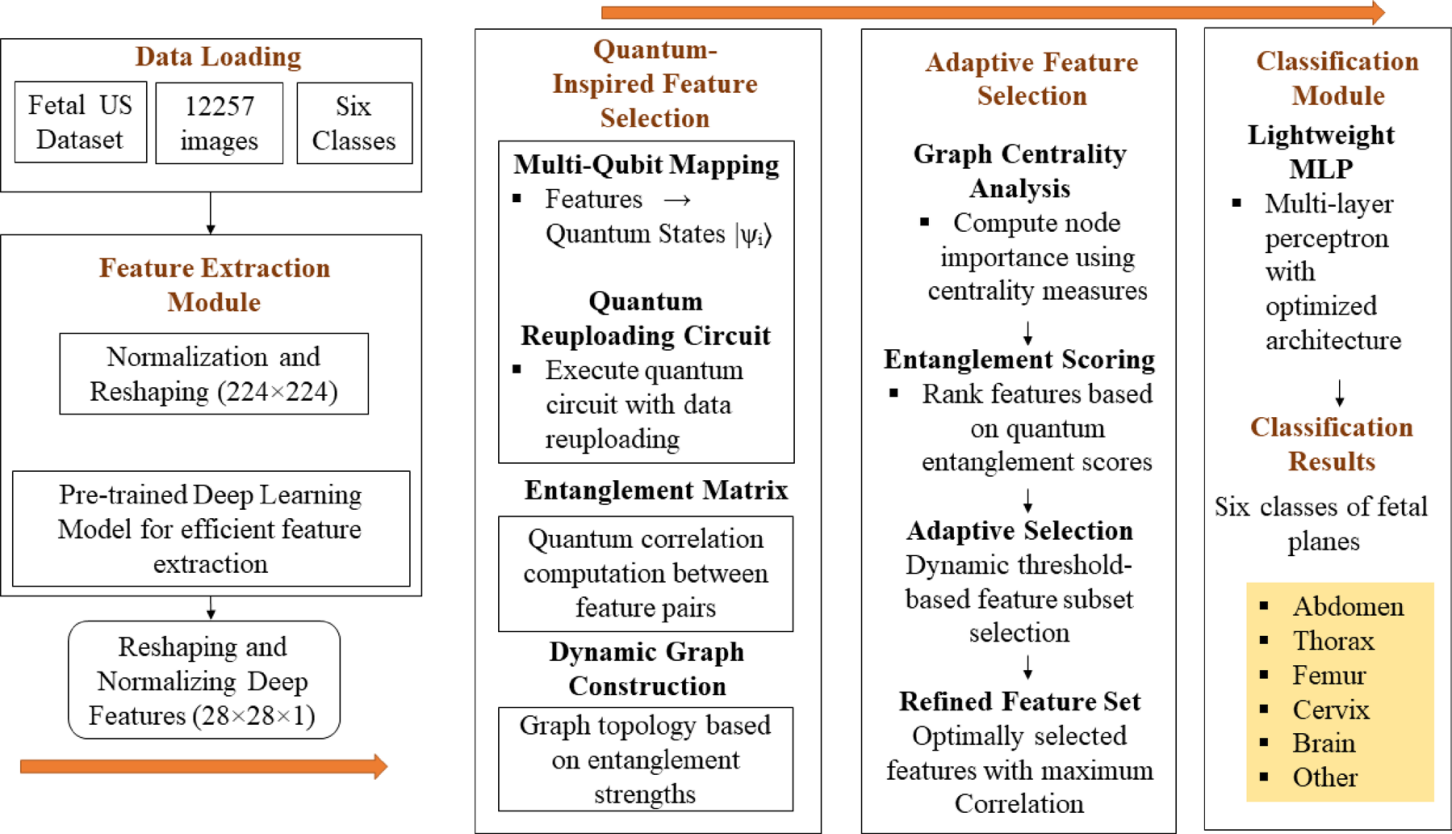
Architecture of the quantum-inspired DG-QFS framework illustrating the interaction between deep features and the parameterized quantum circuit. Extracted deep features are mapped into quantum states with data re-uploading, which are entangled, and processed within a dynamic graph structure for feature selection. Therefore, the figure highlights that quantum entanglement and graph-based refinement jointly enhance the selection of discriminative features.

The framework illustrated in Fig. 3 is a quantum-inspired pipeline for Fetal US classification into six biometric planes. Initially, a pre-trained model extracts deep features from normalized and reshaped input images. These features are mapped to the quantum states and processed using a quantum re-uploading circuit to capture complex dependencies through entanglement. A graph-based adaptive feature selection method ranks and refines features using quantum entanglement scores and centrality measures. Finally, a lightweight MLP classifier predicts the Fetal plane category with enhanced accuracy using the optimally selected features.

### Deep feature extraction

Mobile-Net, a lightweight convolutional neural network based on depth-wise separable convolutions, is employed for deep feature extraction, which is well-suited for medical imaging applications due to its low.

parameter count and high representational efficiency. As demonstrated in Table 7, Mobile-Net outperforms other architectures, resulting in the best performance in feature extraction for the Fetal plane US dataset. Each input US image ∈^××^, R is a set of real numbers consisting of a standard dimension of 224 × 224 × 3, where H, W, and C denote the height, width, and number of channels. The input data are normalized before being inserted into the model. The extracted feature representation is computed as in Eq. (1),

1 $$\left[ {x_{1} ,x_{2} ,...x_{d} } \right]^{T} \in R^{d}$$

Where xi represents the i-th feature component x, T denotes the transpose of the vector, and denotes the dimensionality of the output feature vector in the given input image. Mobile-Net is used without top classification layers, and partially fine-tuned by unfreezing the last 30 layers. Then the outcome is passed through a global average pooling layer, followed by a dense transformation to a 28 × 28 feature map (784-dimensional vector), which is then reshaped to 28 × 28 × 1. This refined output ensures the consistent progress across samples, which helps in stable training for subsequent quantum models.

### Quantum-enhanced feature selection

The proposed method captures intricate feature dependencies by eigenvector centrality and quantum entanglement-based edge weighting in a more efficient way. The hybrid approach outperforms traditional techniques by dynamically refining feature importance using quantum re-uploading circuits. The integration of quantum scores enhances the graph correlation and leads to more informative feature subsets. Therefore, the classifier achieves high accuracy and generalization on complex ultrasound imaging tasks.

#### Initial feature graph construction

High-dimensional US datasets often shows the redundancy due to strong inter-feature correlations. To address this, the proposed model calculates the feature inter-relationships using graph theory. The initial step is to check the pairwise statistical dependencies among features using Pearson correlation coefficients. The dataset ∈ ^×^, where ‘n’ is the number of samples and ‘d’ is the number of features, defines the correlation between two features (and) in Eq. 2 as,

2 $$\:\mathrm{Corr}\left({x}_{i},{x}_{j}\right)=\frac{\mathrm{Cov}\left({x}_{i},{x}_{j}\right)}{{\sigma\:}_{{x}_{i}}\cdot\:{\sigma\:}_{{x}_{j}}}=\frac{E\left[\left({x}_{i}-\stackrel{-}{{x}_{i}}\right)\left({x}_{j}-\stackrel{-}{{x}_{j}}\right)\right]}{\sqrt{E\left[{\left({x}_{i}-\stackrel{-}{{x}_{i}}\right)}^{2}\right]\cdot\:E\left[{\left({x}_{j}-\stackrel{-}{{x}_{j}}\right)}^{2}\right]}}$$

The resulting matrix C_abs_ ∈ R^d×d^ is symmetric and captures the magnitude of linear relationships between features. The Pearson correlation coefficient measures the linear relationship between two feature components, x_i_ and x_j_. It is computed by normalizing the covariance with the product of their standard deviations $\:({\sigma\:}_{{x}_{i}}$.$\:{\sigma\:}_{{x}_{j}})$, ensuring scale independence. It encodes the absolute strength of linear associations between features, and it forms the foundation for graph construction.

#### Adjacency matrix construction with thresholding

The feature correlation is structured as a weighted undirected graph G (V, E), where each node v_i_ ∈ V depicts a feature (x_i ​_ and x_j_). Each edge (v_i_, v_j_) ∈ E carries a weight corresponding to the correlation strength. To eliminate the noisy relationships, a thresholding strategy is used. Let τ ∈ [0,1] be the threshold parameter. The adjacency matrix A ∈ R ^d×d^ is given in Eq. (3) as,

A_ij_=$\:{\{}_{0,}^{\mid\:Corr\:({x}_{i},{x}_{j})\mid\:}$ If ∣Corr (x_i_, x_j_)∣ > τ and i$\:\ne\:$j.

3 $$\begin{aligned} A_{{ij}} = \left\{ {_{{0,}}^{{\left| {Corr\left( {x_{i} ,x_{j} } \right)} \right|}} } \right.If & \left| {Corr\left( {x_{i} ,x_{j} } \right)} \right|> \tau & and & & i \ne j \\ Otherwise \\ \end{aligned}$$

The above Eq. (3) represents the connections between features denoted as d ' in the adjacency matrix. Each entry $\:{A}_{ij}\:$is the absolute value of the correlation $\:\mid\:\mathrm{Corr}\left({x}_{i},{x}_{j}\right)\mid\:\:\:$if it exceeds a threshold $\:\tau\:$, which is a predefined value used to filter out unwanted correlations. If $\:\mid\:\mathrm{Corr}\left({x}_{i},{x}_{j}\right)\mid\:\le\:\tau\:\:,\:$the entry is set to 0, and ensures strong relationships are captured in the graph. Therefore, it enhances the computational efficiency and interpretability of the resulting graph.

#### Quantum feature state encoding

To enrich the feature correlation analysis beyond classical metrics, a quantum-enhanced mechanism based on entanglement scores derived from quantum circuits is proposed. Entanglement is a quantum effect where each qubit becomes interdependent such that the state of one cannot be described independently of the others. The algorithm enables the capture of non-classical, high-order dependencies between features that are often missed by conventional correlation-based graphs.

##### Quantum state mapping and circuit encoding

The set of selected features, map them into the quantum states using angle encoding. To enhance the expressivity of the quantum circuit, adopt a quantum re-uploading strategy, where classical data is encoded into the circuit at multiple layers or depths.

The data is encoded as rotation angles in a parameterized quantum circuit in Eq. (4) as,

4 $$U_{{encode}} (x_{i} ,x_{j} ){\text{ }} = x_{i} \otimes x_{j}$$

5 $$U_{{en\tan gle}} = {\text{ }}CNOT{\text{ }}i \to j{\text{ }}or{\text{ }}CZ_{{i,{\text{ }}j}}$$

**Fig. 4:**
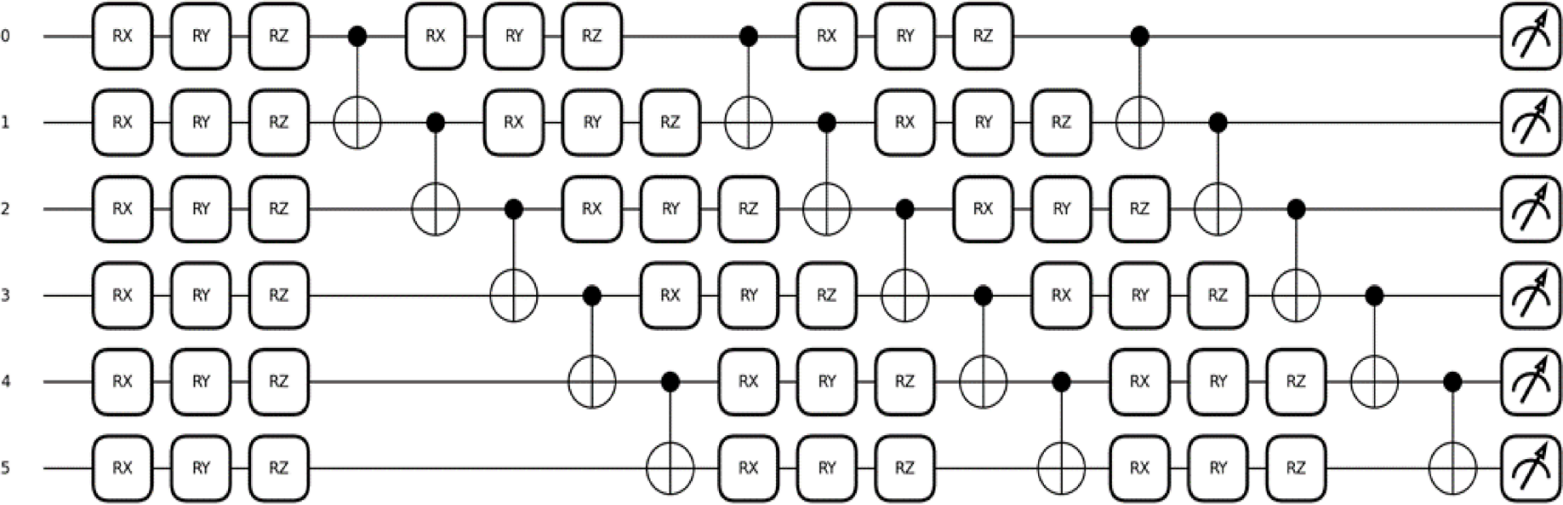
Multi-qubit parameterized quantum circuit with entanglement layers used in the DG-QFS framework. To model the complex features, a quantum circuit initialized with 6 qubits are entangled through CNOT gates, and it is used to capture non-linear correlations.

The quantum feature encoding process shown in Eq. (4) has classical features _i_ and, which are mapped into a joint quantum state using the tensor product. It allows the features to exist simultaneously in a higher-dimensional quantum space. Equation (5) is applied to introduce entanglement between the qubits by linking their states (i and j). The CNOT gate flips the target qubit conditional on the control qubit, while the CZ gate applies a phase flip if the control qubit is ∣1⟩. It enables the quantum circuit, which is depicted in Fig. 4, to capture both detailed feature information and its correlations in a more accurate way.

##### Quantum Re-uploading for iterative data embedding

To improve feature representation and allow the circuit to model complex relationships, apply the classical inputs across multiple layers of the quantum circuit, interleaved with trainable parameter gates. Each layer has the mathematical form as shown in Eq. (6),

6 $$U^{{\left( l \right)}} \left( {x_{i} ,x_{j} } \right) = \:U_{{en\tan gle}}^{{\left( l \right)}} \:U_{{encode}}^{{\left( l \right)}} \:\left( {x_{i} ,x_{j} } \right)U_{{param}}^{{\left( l \right)}}$$

where:

- Inputs are re-encoded at each layer.
- $\:{\mathrm{U}}_{\:\mathrm{e}\mathrm{n}\mathrm{c}\mathrm{o}\mathrm{d}\mathrm{e}\:}^{\left(\mathrm{l}\right)}$ , embeds the input features at layer.
- $\:{\mathrm{U}}_{\mathrm{p}\mathrm{a}\mathrm{r}\mathrm{a}\mathrm{m}\:\:}^{\left(\mathrm{l}\right)}$is a trainable block of gates (e.g., , ),
- $\:{\mathrm{U}}_{\mathrm{e}\mathrm{n}\mathrm{t}\mathrm{a}\mathrm{n}\mathrm{g}\mathrm{l}\mathrm{e}}^{\left(\mathrm{l}\right)}$ Introduces entanglement by CNOT gates across qubits.

The complete circuit across L layers is expressed as a product of unitary blocks is given in Eq. (7) as,

7 $$(x_{i} ,x_{j} ) = \prod _{{(l = 1)}}^{L} U(l)(x_{i} ,x_{j} )$$

The above Eq. (7) deep re-uploading circuit structure allows the model to express highly nonlinear dependencies between features. It represents the sequential application of unitary operations on input features $\:({x}_{i},{x}_{j})$across $\:{L}^{{\prime\:}}\:$layers. Here, $\:{U}^{\left(l\right)}\:$denotes the unitary operator at the $\:{l}^{th}\:$layer, and the symbol $\:\prod\:\:\:$combines the transformations.

##### Entanglement score-based feature ranking

After executing the quantum circuit, extract an entanglement score to quantify the dependency between feature-representing qubits and rank the features based on quantum entanglement scores to identify the most informative feature nodes. The Von Neumann Entropy and Concurrence metrics are used to calculate the quality of the entanglement score are listed below.

(a) Von Neumann Entropy: Let ρ be the final state density matrix, and ρ_i_=Tr(ρ) be the reduced density matrix of qubit. The entanglement entropy is given in Eq. (8),

8 $$E_{{ij}} = {\text{ }} - Tr{\text{ }}(\rho _{i} \log \rho _{i} )$$

Equation (8), E_ij_ represents entanglement entropy, where Tr denotes the trace of the matrix and ρ_i_ is the reduced density matrix of subsystem.

(b) Concurrence: As an alternative entanglement measure for pure states, the concurrence is computed as,

9 $$C{\text{ }}(\rho ){\text{ }} = {\text{ }}\max {\text{ }}(0,\lambda _{1} - \lambda _{2} - \lambda _{3} - \lambda _{4} )$$

Equation (9) represents the concurrence (ρ) is an entanglement measure computed using the eigenvalues of the matrix in decreasing order. In Eq. (10) below, where is the Pauli-Y matrix, ⊗ denotes the tensor product, and ρ∗ is the complex conjugate of the density matrix.

10 $$R = {\text{ }}(\sigma _{Y} \otimes \sigma _{Y} )\rho ^{ * } (\sigma _{Y} \otimes \sigma _{Y} )$$

**Fig. 5:**
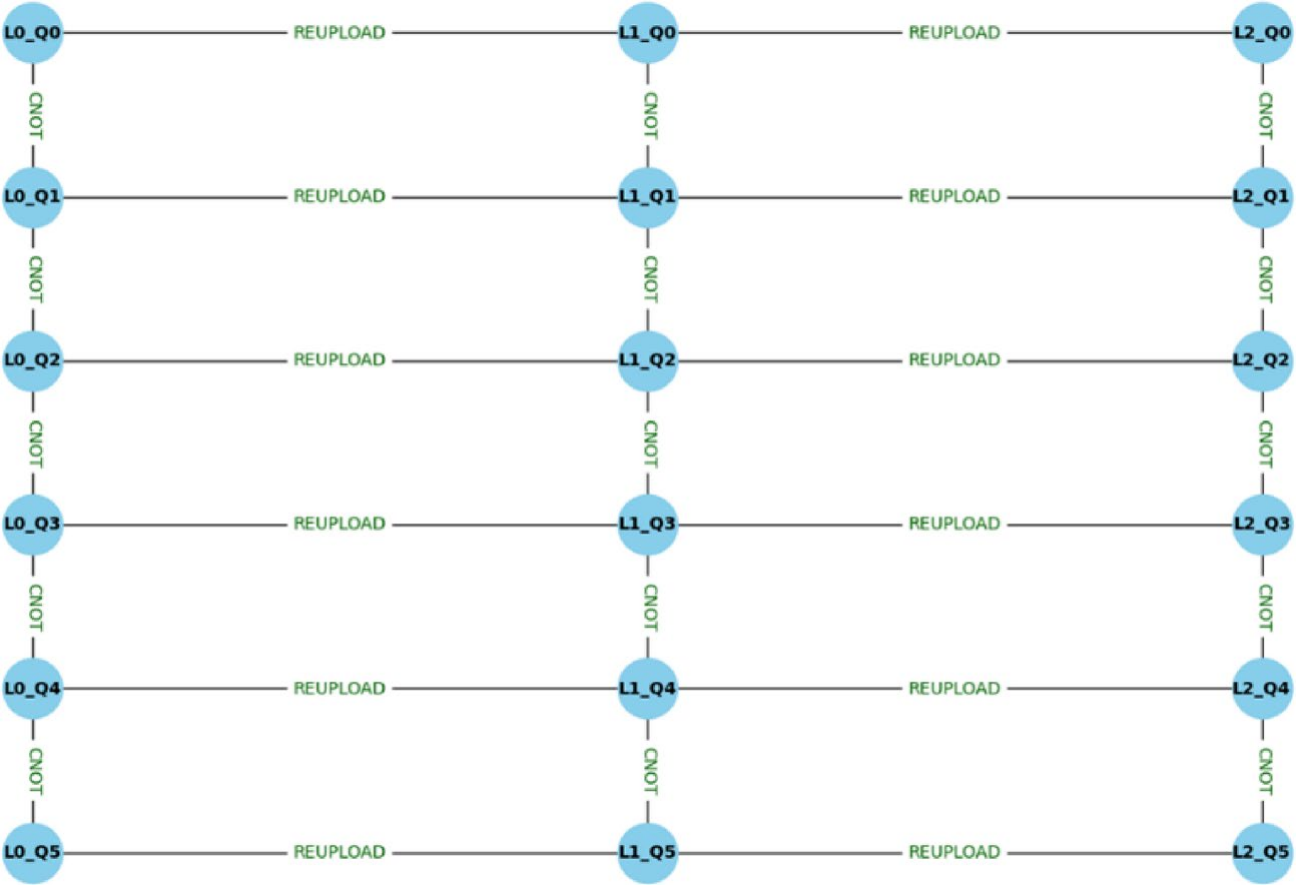
Quantum re-uploading nodal circuit architecture depicts the iterative data encoding across multiple layers. Classical features are re-embedded at each layer and passed through trainable gates, which improves the feature discriminability for fetal classification tasks.

**Fig. 6:**
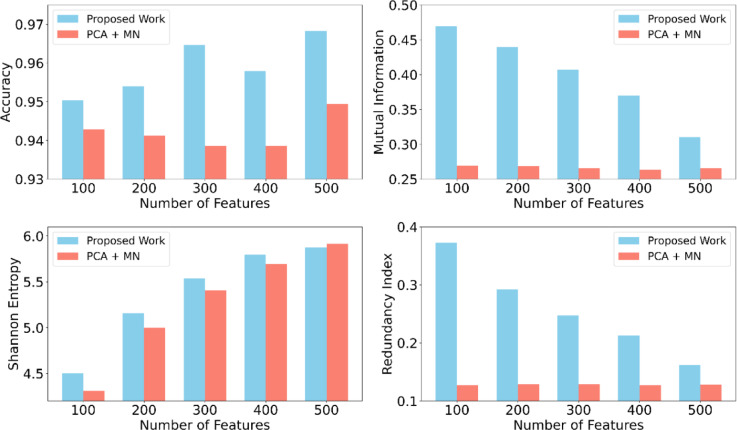
Performance comparison between the proposed DG-QFS and PCA + Mobile-Net techniques across different feature counts (from 100 to 500). The bar plot shows that the DG-QFS consistently achieves higher accuracy and mutual information with lower redundancy values.

The above metrics provide a scalar score ∈ [0,1] indicating the strength of quantum correlation. Figure 5 depicts a quantum re-uploading circuit architecture using six qubits across three layers. Each layer applies a data re-uploading process by encoding classical features into quantum states, and it is then connected with CNOT gates to entangle the qubits vertically across layers. The resultant structure enables iterative entanglement and richer quantum feature representation for downstream tasks such as feature selection and classification, Fig. 6.

##### Dynamic edge weight refinement by entanglement scores

The entanglement score is computed for all feature pairs (, ) and dynamically updates the edge weights in the feature graph, as given in Eq. (11),

11 $$w_{{ij}} (t + 1) = w_{{ij}} (t) + (1 - \beta ) \cdot E_{{ij}}$$

where is the current edge weight, is the quantum entanglement score, ∈ [0,1] is the fidelity factor, balancing classical correlation and quantum-enhanced information. The update rule ensures that the feature graph evolves in a data-driven manner, influenced by both classical metrics and non-classical metrics. Therefore, the high-order dependencies are captured by the quantum entanglement circuit with re-uploading, as shown in Fig. 5.

#### Graph centrality-based feature importance Estimation

Eigenvector centrality is a measure of the influence of a node in graph theory, not based on its direct connections, but also on the importance of its neighbors. The adjacency matrix, the eigenvector centrality vector ∈ ^d^ is the principal eigenvector satisfying the Eq. (12), as given below,

12 $$Ac = \lambda c$$

Where is the largest eigenvalue of A. Each component denotes the relative importance of feature within the feature graph. The spectral property captures both local and global connectivity, which is used to rank and select the top-*k* most influential features. In practice, eigenvector centrality offers a more robust alternative to degree centrality or mutual information when features interact in complex, transitive ways.

#### Iterative graph refinement

The overall feature graph is iteratively refined in a loop and combines the updated edge weights and recalculates centralities by the given rule below,

- Update edge weights using quantum entanglement scores.
- Recompute centralities and feature importance rankings.
- Reselect top- features.
- Retrain or fine-tune the classifier with selected features.

Let the time-dependent graph, G (t) = (, (t)), where V is the fixed set of vertices and E (t) is the set of edges with iteration t, and let C (t) denote the eigenvector centrality vector. Then, the refinement process can be expressed recursively as in Eq. (13),

13 $$G{\text{ }}\left( {t + 1} \right){\text{ }} = {\text{ }}Update{\text{ }}Edges\left( {G\left( t \right),{\text{ }}Q} \right)$$

14 $$C (t+1) = Eigenvector Centrality(G(t+1))$$

Here, G(t + 1) denotes the graph at time (t + 1), after updating its structure. Above Eq. (14) continuously updates the edge weight of the graph at time t by the structural update rule Q. C(t + 1) is obtained using eigenvector centrality, which measures the importance of each node based on the influence of its neighbors in the updated graph. The above iterative loop allows the system to converge towards a stable set of optimal features.

#### Final edge pruning strategy

The Weak edges are pruned after each iterative update to ensure sparsity and avoid overfitting. Specifically, for an edge (, ), if the updated weight falls below the threshold τ, or is equal to zero. It is illustrated in Eq. (15) below as,

15 $$If~~~~A_{{ij}} ^{{new}} < \tau ,{\text{ }}then{\text{ }}Aij^{{new}} = {\text{ }}0$$

This dynamic pruning promotes a sparse, interpretable, and high-quality sub-graph, enabling more efficient feature selection and improving model generalizability.

### Optimized MLP classifier for fetal ultrasound image classification

The performance of selected features is tested by a Multi-Layer Perceptron (MLP), a lightweight neural classifier that operates with an optimized architecture, as shown in Table 2. The Input features are standardized by the StandardScaler technique to achieve zero-mean and unit-variance scaling. The MLP model used three hidden layers comprising 256, 128, and 64 neurons, respectively, and utilizes the ReLU activation function. Train by stochastic gradient descent (SGD) optimizer with an adaptive learning rate strategy, with an initial rate of 0.01, and L2 regularization with alpha set to 0.0005. The training procedure incorporates early stopping, monitoring validation performance using 10% of the training data, and halting when no improvement appears over 10 consecutive iterations. Following training, accuracy and comprehensive classification metrics are computed on the test set to robustly assess the effectiveness of the learned feature representations..

**Table 2:** Hyperparameters of the MLP classifier.

Parameter	Value
Hidden layers	(256, 128, 64)
Activation function	ReLU
Optimizer	SGD
Learning rate strategy	Adaptive
Initial learning rate	0.01
Regularization (alpha)	0.0005
Batch size	64
Maximum iterations	500
Early stopping	Enabled
Feature scaling	StandardScaler

**Table 3:** Evaluation metrics and their formulas.

Metric	Formula
Accuracy	$((TP + TN) )/((TP + TN + FP + FN))$
Precision	$(TP )/((TP + FP ))$
Recall (Sensitivity)	$(TP )/((TP + FN ))$
F1-Score	$\frac{{2 \times (\Pr ecision \times \mathrm{Re} call)}}{{(\Pr ecision + \mathrm{Re} call)}}$
Mutual Information (MI)	$\sum _{{x \notin X}} \sum _{{y \notin Y~}} p\left( {x,y} \right) \log _{2} \frac{{p\left( {x,y} \right)}}{{p(x) \times p(y)}}$
Shannon Entropy	$H(X){\text{ }} = {\text{ }} - \sum {p(x_{i} ){\text{ }}\log 2(p(x_{i} ))} {\text{ }}$
Redundancy Index (RI)	$`RI = 2/(n(n - 1))\sum\nolimits_{{(i = 1)}}^{n} {...} \sum\nolimits_{{(j = i + 1)}}^{n} {} |rij|$

## Results and discussion

The experimental setup for the classification of fetal US planes using a large, publicly available dataset consists of annotated details with six anatomical categories, and is carried out in Jupyter Notebook on the Anaconda platform, utilizing a high-performance system with a 64GB GPU. This implementation includes Python 3.7 version along with key libraries such as Pennylane 0.37 and TensorFlow 2.17. Feature extraction is performed using a pre-trained Mobile-Net model, after which the deep features are refined using a novel Multi-Qubit Entanglement-Based Dynamic Graph Feature Selection method. The proposed DG-QFS method combines quantum entanglement scores and graph node structure with centrality analysis to select the most relevant features. The optimized features are given to the MLP classifier to predict the corresponding fetal plane with high accuracy. The model’s performance is evaluated using standard classification metrics and feature selection metrics with several cycles to ensure reliability and suitability for high-dimensional data.

### Evaluation metrics

The proposed Multi-Qubit Entanglement-Based Dynamic Graph Feature Selection DG-QFS framework is designed to improve the classification of fetal US images into six anatomical classes. To test the performance analysis of the proposed method, several comprehensive metrics were employed. Accuracy, precision, recall, and F1-score are used to assess the classification capability of the lightweight MLP classifier in predicting the correct fetal plane classes by measuring their True Positive (TP), True Negative (TN), False Positive (FP), and False Negative (FN).

In addition to that, Mutual Information (MI) is used to quantify the importance of selected features to their target labels and ensure that the most informative features are retained for the classification task.The Shannon Entropy is used to evaluate the diversity of the feature by offering insights into the selected information. To monitor the duplication in features, the Redundancy Index (RI) is calculated to identify the correlated features that do not contribute to the classification task. The metrics are listed in Table 4 and collectively validate the proposed system’s robustness in feature selection and classification and confirm its ability to maintain high performance while reducing feature dimensionality.

### Key findings

The key findings are performance metrics for classification, which evaluate the overall effectiveness of the model using accuracy, precision, recall, and F1-score. Feature Selection evaluation examines the quality of the selected features using metrics like mutual information, Shannon entropy, and redundancy index. The Performance Analysis of the Proposed DG-QFS shows the model’s stability, convergence behavior, and its superiority over baseline methods. Moreover, this section presents the Class-wise Metrics and Quantum Entanglement Insights, which in turn explain a deep understanding of per-class performance and the role of quantum entanglement in feature selection.

#### Classification performance metrics

**Table 4:** Classification accuracy comparison.

Model	Accuracy (%)	Precision (%)	Recall (%)	F1-Score (%)
Mobile-Net (No Feature Selection)	94.09	94.44	94.09	94.02
Mobile-Net + PCA + MLP	95.52	95.57	95.52	95.51
Mobile-Net + Mutual Information + MLP	93.01	93.42	93.01	93.13
Mobile-Net Lasso Feature Selection + MLP	95.13	95.19	95.13	95.15
Mobile-Net + QAOA + MLP	90.17	90.43	90.17	90.25
Auto encoder-Based Feature Selection	93.60	93.72	93.60	93.61
Proposed (DG-QFS)	**96.73**	**96.87**	**96.70**	**96.67**

Table 4 compares various feature selection and classification approaches for US image analysis, with the proposed DG-QFS (Dynamic Graph-based Feature Selection) model, by achieving enhanced performance across all metrics. (DG-QFS) shows the prominent improvement by achieving 96.70% accuracy, 96.87% precision, 96.70% recall, and a 96.67% F1-score. The proposed model provides parameterized entanglement and dynamic graph structures to capture both local and global dependencies in the feature space, enabling the selection of the most relevant and non-redundant features. Unlike traditional methods, it dynamically adapts to the data distribution and models complex inter-relationships using quantum circuits and graph-based learning, and contributes to its superior performance. Mobile-Net (baseline model), without any feature selection, obtains a score of 94.09% in accuracy and results in lower performance compared to the proposed method, due to the presence of redundant and noisy features. The Mobile-Net + Principal Component Analysis (PCA) with MLP classifier model improves accuracy to 95.52% by reducing dimensionality through linear transformations that preserves a maximum variance. But the PCA model fails to capture complex non-linear correlations among features and degrades its discriminative power. Extracting the deep features using a pre-trained Mobile-Net architecture is combined with Mutual Information (MI) for selecting important features. Further is classified by the MLP algorithm, is lags with an accuracy of 93.01%, as MI selects features based on pairwise relevance with the class label, but ignores multi-feature interactions and contextual information, and it is less effective for structured image data. The Mobile-Net + Lasso Feature Selection method shows better performance with 95.13%, using L1 regularization to insert sparsity in the feature set. While the Lasso effectively removes less informative features, and assumes that the linear relationships may discard valuable correlated features. Mobile-Net + QAOA (Quantum Approximate Optimization Algorithm) + MLP records the lowest performance at 90.17% accuracy.

The sub-optimal optimization in the quantum feature selection process limited the entanglement operation. Finally, the Auto autoencoder-based feature selection performs moderately well with 93.60% accuracy by learning compact representations, but fails to prioritize class-discriminative features. Overall, the DG-QFS approach outperforms all other methods due to its ability for dynamically model feature dependencies and harness quantum entanglement, resulting in better generalization and classification performance in complex medical imaging tasks.

#### Feature selection evaluation metrics

**Table 5:** Comparative performance analysis: proposed feature selection method vs. PCA + MN Baseline.

Features	Accuracy (Proposed Work)	Accuracy (PCA +MN)	MI (Proposed work) (bits)	MI (PCA +MN) (bits)	Entropy (Proposed work) (bits)	Entropy (PCA +MN) (bits)	RI (Proposed work)	RI (PCA +MN)	FS Time (Proposed work) (s)	FS Time (PCA +MN) (s)	Train Time (Proposed work) (s)	Train Time (PCA +MN) (s)	Inference Time (Proposed work) (s)	Inference Time (PCA +MN) (s)
100	**0.9503**	0.9428	**0.4692**	0.2694	**4.5016**	4.3090	0.3726	0.1268	660.01	**0.27**	45.16	**29.50**	0.0117	0.01
200	**0.9539**	0.9412	**0.4393**	0.2685	**5.1540**	4.9959	0.2924	0.1285	645.09	**0.35**	43.18	**27.72**	0.0098	0.01
300	**0.9647**	0.9386	**0.4070**	0.2654	**5.5356**	5.4025	0.2470	0.1289	669.28	**0.39**	48.31	**33.86**	0.0127	0.01
400	**0.9579**	0.9386	**0.3699**	0.2634	**5.7946**	5.6953	0.2126	0.1271	674.93	**0.56**	55.89	**39.55**	0.0242	0.02
500	**0.9673**	0.9494	**0.3104**	0.2654	5.8760	5.9155	0.1618	0.1278	664.88	**0.47**	61.48	**40.48**	0.0184	0.02

Table 5 shows the performance of the proposed DG-QFS method against PCA + Mobile-Net + MLP (PCA + MN), which is chosen as a strong baseline from the best-performing classification models from Table 3. The analysis consists of key metrics across feature subset sizes (100 to 500), including classification accuracy, mutual information (MI), Shannon entropy, redundancy index (RI), feature selection time (FS Time), training time, and inference time. From the different feature sizes, the proposed method consistently outperforms the PCA + MN model in terms of accuracy, peaking at 96.73% for 500 features, whereas PCA + MN scored 94.94% for 500 features as depicted in Fig 6. MI and RI, indicating more informative and less redundant feature subsets. From the analysis, the FS time is longer due to the complexity of DG-QFS, and the trade-off results in superior accuracy and generalization. The inference time remains comparable and ensuring real-time feasibility. Overall, highlights the efficiency and robustness of the proposed strategy over conventional dimensionality reduction methods. Although the PCA + MN model demonstrates a lower RI compared to the proposed DG-QFS method, its accuracy remains lower because PCA reduces dimensionality by projecting features onto orthogonal components with the highest variance; it may discard the class-discriminative features during the transformation. In contrast, DG-QFS selects features based on quantum-inspired entanglement and mutual dependencies from the dynamic graph nodal architecture that are directly relevant to classification tasks and preserves both the informative and class-relevant features. As a result, despite having slightly higher redundancy, DG-QFS achieves higher accuracy by maintaining a more discriminative feature set tailored for the target labels.

#### Performance analysis of proposed (DG-QFS)

**Fig. 7:**
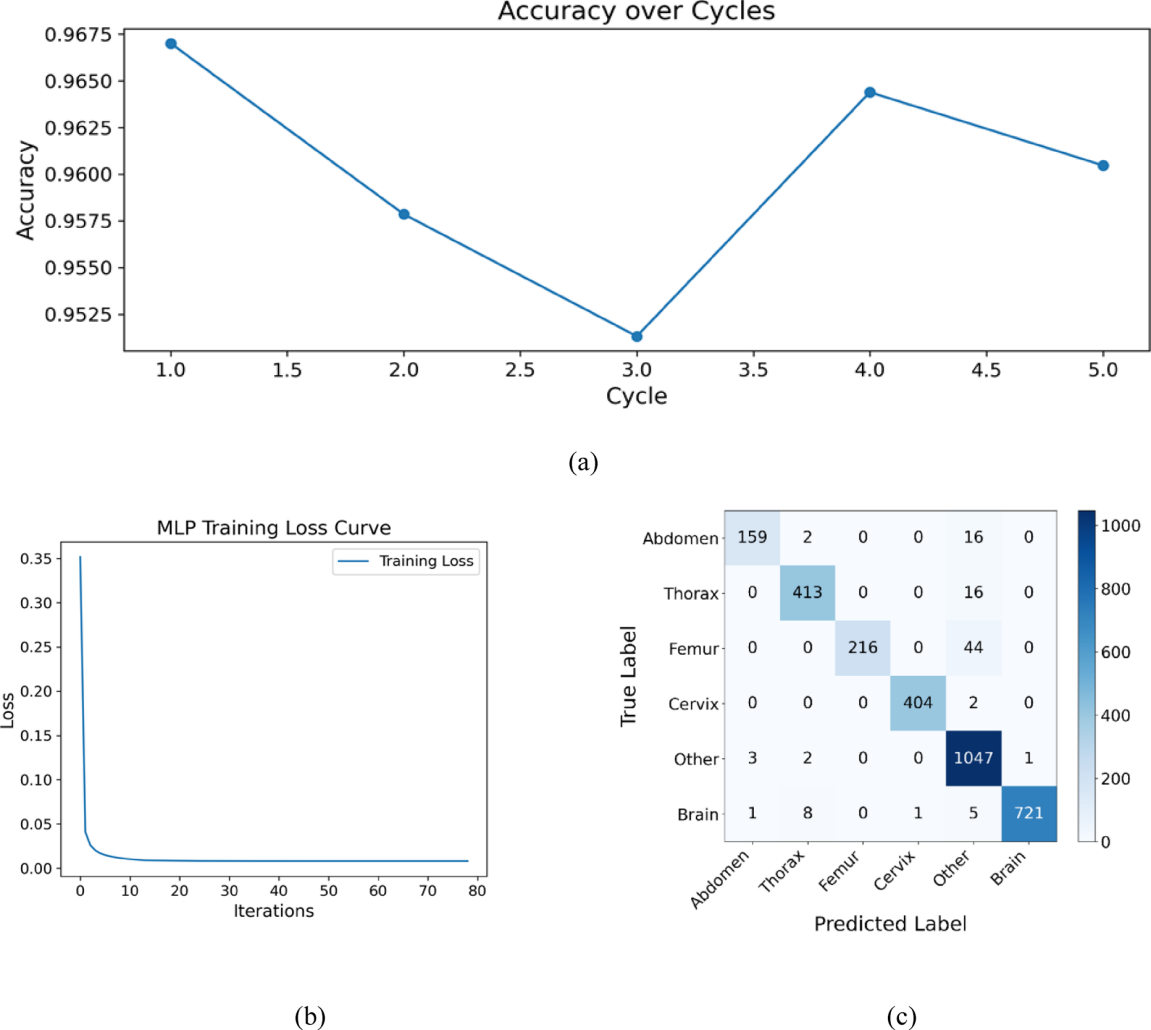
Performance analysis of the DG-QFS framework. (**a**) Classification accuracy remains stable across five quantum feature selection cycles, which indicates consistent feature selection. (**b**) Training loss curve for MLP classifier with DG-QFS features resulting in smooth convergence. (**c**) Confusion matrix showing class accuracy across all six fetal anatomical categories.

The following section evaluates the effectiveness of the DG-QFS model using class-wise performance metrics, its convergence behavior, and quantum entanglement analysis. The Fig. 7a shows that the model maintains high classification accuracy (above 95%) over five feature selection cycles, with only minor fluctuations, and indicates the consistent performance despite iterative feature refinement. Figure 7b reveals a rapid and smooth convergence of the model, with the training loss dropping sharply within the initial iterations and stabilizing near zero, signifying effective learning and minimal over-fitting. The Fig. 7c show the confusion matrix has strong classification performance across all six anatomical classes, with especially high true positive counts for critical categories like “Other” (1040), “Brain” (722), and “Cervix” (405), while maintaining low misclassification rates, thus confirming the model’s robustness and reliability in multi-class medical image classification tasks.

**Table 6:** Per-class performance ((DG-QFS)).

Class	Precision	Recall	F1-Score
Abdomen	0.9755	0.8983	0.9353
Thorax	0.9718	0.9627	0.9672
Femur	1.0000	0.8308	0.9076
Cervix	0.9975	0.9951	0.9963
Other	0.9265	0.9943	0.9592
Brain	0.9986	0.9796	0.9890

The class-wise performance metrics are in the Table 6 shows the DG-QFS model proves its strong generalization and discriminative capability across diverse fetal anatomical structures. Notably, the Cervix and Brain classes achieved exceptionally high precision (99.75% and 99.86%) and F1-scores (99.63% and 98.90%), indicating highly reliable and consistent predictions. Class “Thorax” shows balanced and high values among all metrics, and it reflects robust detection. The class “Femur” has a perfect precision of 100% but a relatively lower recall of 83.08%.

This suggests the model is conservatively accurate when it is predicting” Femur,” but misses some true cases. Class” other “exhibited the maximum recall (99.43%) but minimum precision (92.65%), and it captures the true instances at the cost of some false positives. The “Abdomen” class shows the lowest recall (89.83%) and a moderate F1-score (93.53%), indicating the room for improvement in detecting all relevant samples. On comparing with all models, the proposed DG-QFS design delivers a maximum and consistent accuracy across six classes.

**Fig. 8:**
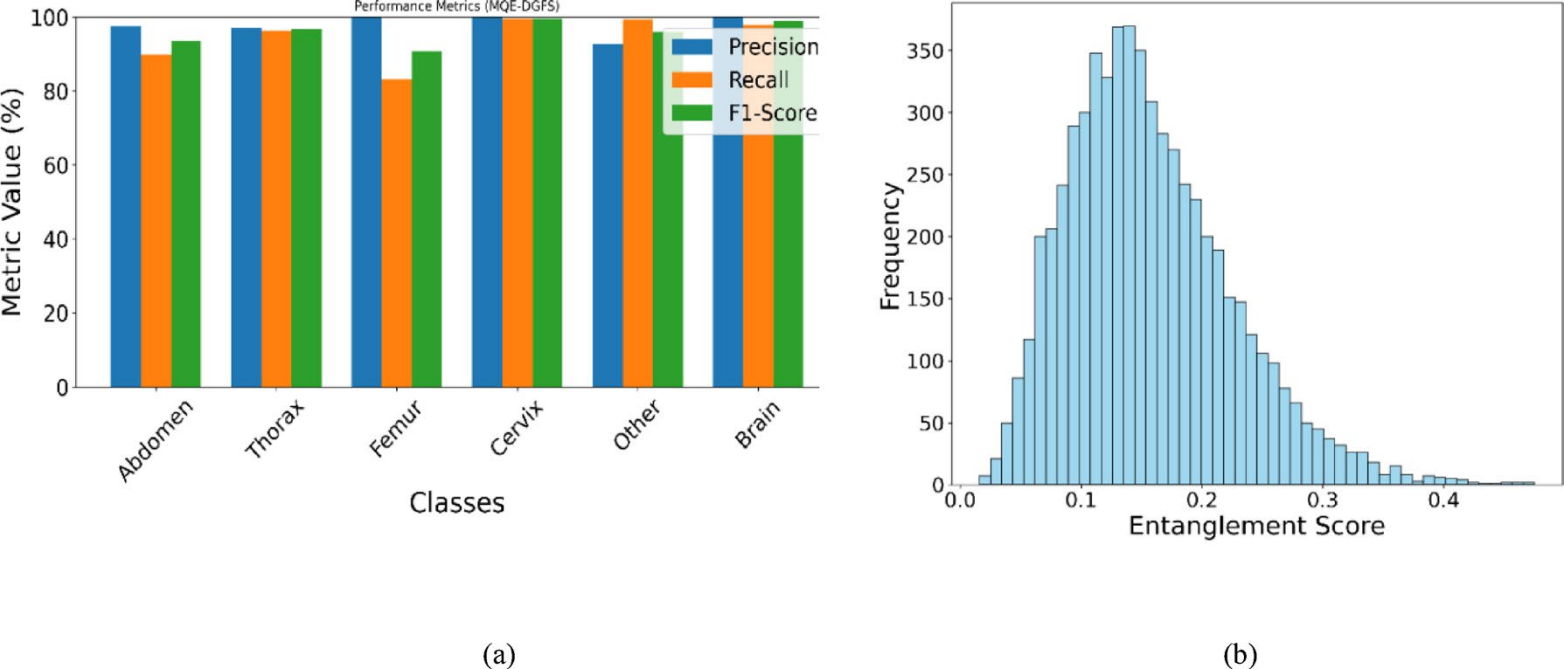
(**a**) Class-wise evaluation metrics for the DG-QFS method show the consistent performance across all fetal US plane classes, with Brain and Cervix achieving near-perfect values. (**b**) The entanglement score distribution results that entangled features from (0.1–0.2) dominate the feature space, contributing to discriminative performance in classification.

Figure 8a represents the bar chart, which shows the precision, recall, and F1-score for six fetal ultrasound classes using the DG-QFS model. The model performs consistently well across all classes, with the cervix and brain classes achieving near-perfect scores. Figure 8b illustrates the histogram distribution of entanglement scores derived from the process of quantum feature selection process. The scores are mostly between 0.1 and 0.2, which indicates that moderate quantum entanglement dominates the feature space. The distribution is right-skewed, with fewer features and exhibits more entanglement beyond the value 0.3. It suggests that the highly entangled features exist, but are relatively small and represent complex correlations. The histogram highlights that informative features are primarily associated with the moderate levels of quantum correlation.

### Discussion

The evaluation outcomes from Tables 2 and 4 show the superior performance of the proposed DG-QFS model in US plane classification and demonstrate the effectiveness of integrating quantum entanglement with dynamic graph-based feature selection. Unlike the conventional techniques such as PCA, Lasso, or mutual information, DG-QFS adaptively models all dependencies in the feature space through parameterized quantum circuits, resulting in higher accuracy (96.73%) and balanced precision, recall, and F1-scores across all classes. The class-wise performance confirmed the robustness of the model and especially for critical anatomical categories like the Brain and Cervix, which achieved near-perfect precision and recall. The conservative prediction behavior is observed in the Femur class, and the high sensitivity but lower precision in the other class indicates areas for potential refinement. Notably, the entanglement histogram distribution shows that informative features are mostly associated with moderate quantum entanglement, suggesting an important spot for balancing complexity and relevance in quantum-enhanced feature extraction. Therefore, the findings demonstrate that DG-QFS is not only effective in boosting classification performance but also offers interpretability in how quantum entanglement contributes to feature relevance.

#### Ablation study

An ablation study is to assess the better performance of pre-trained deep learning models and the combined contributions of graph-based, quantum, and random feature selection methods toward ultrasound image classification performance.

**Table 7:** Performance comparison of pre-trained deep learning models for the DG-QFS model.

Model	Accuracy (%)	Precision (%)	Recall (%)	F1 score (%)	Specificity (%)	Kappa score (%)	MCC
VGG-19	94.15	94.30	94.15	94.12	98.65	92.41	0.92
Resnet-50	87.74	86.42	89.50	86.98	97.54	84.50	0.84
Densenet-121	92.42	92.85	92.42	92.54	98.36	90.26	0.90
Mobile-net	**94.34**	**94.55**	**94.34**	**94.42**	**98.77**	**92.72**	**0.92**
Inception -v3	90.75	89.01	89.99	89.31	98.05	88.14	0.88

**Table 8:** Ablation study on feature selection strategies.

Configuration	Accuracy (%)	Precision (%)	Recall (%)	F1-score (%)
Graph-based feature selection	94.90	95.03	94.90	94.95
Quantum feature selection	94.77	94.88	94.77	94.76
Random feature selection	94.94	94.95	94.94	94.92
Mobile-Net + MLP (baseline)	94.09	94.44	94.09	94.02
Proposed (DG-QFS)	96.73	96.87	96.70	96.67

Table 7 presents a comparative analysis of five pre-trained deep learning models, such as VGG-19, ResNet-50, DenseNet-121, Mobile-Net, and Inception-v, across multiple evaluation metrics for a classification task. The lightweight Mobile-Net model obtains the highest accuracy (94.34%), precision (94.55%), recall (94.34%), F1 score (94.42%), specificity (98.77%), Kappa score (92.72%), and MCC (0.92), resulting in the most consistent and reliable model. The Mobile-Net backbone performs slightly better than the VGG-19 model, while DenseNet-121 also shows a lesser performance after Mobile-Net and VGG-19. Inception-v3 and ResNet-50 models have comparatively low performance metrics. Among them, ResNet-50 resulted in the least accuracy compared with all models.

The calculated metrics clearly highlight the Mobile-Net’s superior ability in classification while minimizing false positives and false negatives, and making it particularly well-suited for medical imaging applications. As shown in the Table 8, the proposed (DG-QFS) method significantly outperforms all variants, achieving the highest accuracy (96.73%), precision (96.87%), recall (96.70%), and F1-score (96.67%). The graph-based and quantum feature selection methods yielded comparable results (94.90% and 94.77% accuracy, respectively), indicating each technique is valuable to distinct aspects of feature relevance. The random feature selection results in slightly higher accuracy (94.94%) than quantum-based selection, likely due to incidental preservation of informative features.

**Fig. 9:**
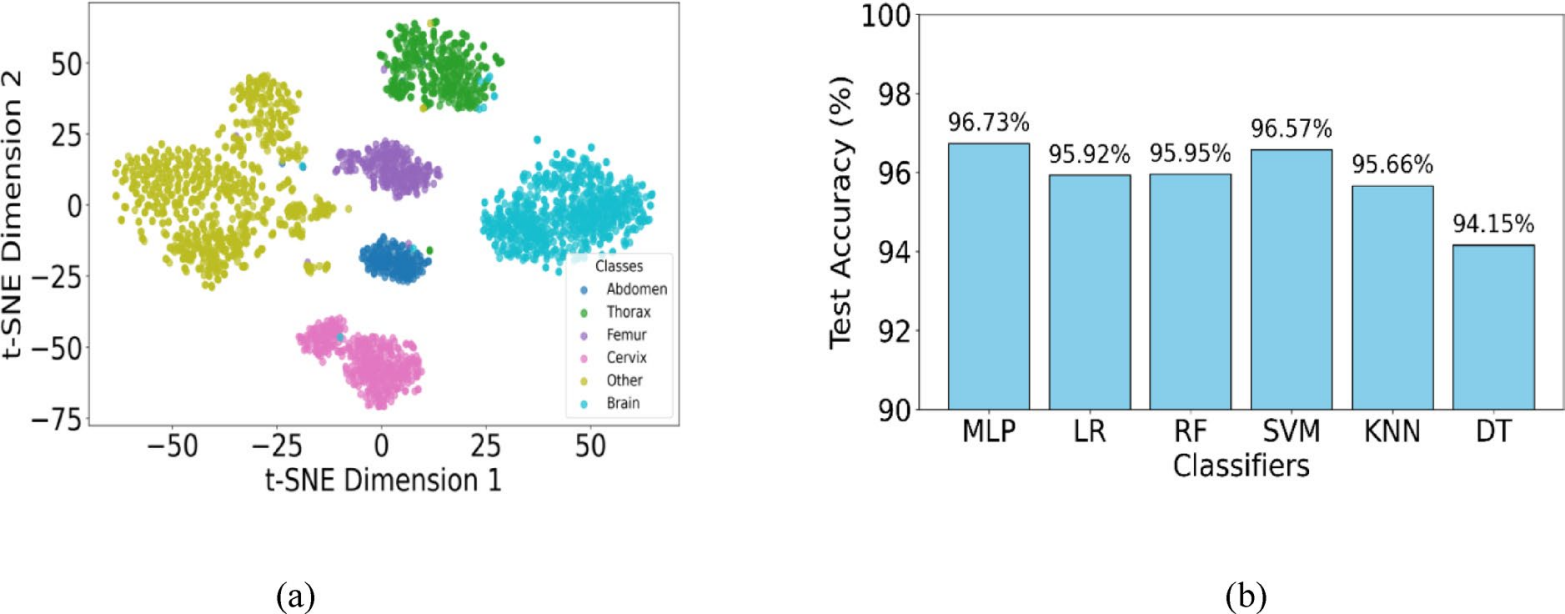
(**a**) t-SNE plot proposed DG-QFS model illustrates clear class separability among six fetal US plane categories and demonstrates strong discriminative feature representation. (**b**) Classifier performance comparison showing that the MLP trained on proposed DG-QFS features outperforms other classifiers.

The baseline model with classification (Mobile-Net + MLP without feature selection) attained the lowest performance (94.09%), demonstrating the critical role of feature refinement. Overall, the proposed study confirms that the integration of quantum entanglement with adaptive graph learning in DG-QFS leads to superior feature selection and classification effectiveness. Figure 9a shows a t-SNE plot of clear separation of the six fetal anatomical classes- Abdomen, Thorax, Femur, Cervix, Other, and Brain class-indicating that the features selected by DG-QFS retain strong discriminative power across complex categories. Figure 9b clearly shows the comparison of MLP classifier performance with other classifiers, where the MLP achieved a high test accuracy of 96.73%, outperforming SVM (96.57%), Random Forest (95.95%), and others. The obtained results confirmed that the proposed DG-QFS design enhances feature separability and improves classification performance when paired with an appropriate classifier.

**Table 9:** Comparison of the proposed model with existing works.

Ref	Accuracy (%)	Precision (%)	Recall (%)	F1-score (%)
^7^	95.69	94.02	96.28	95.08
^8^	91.03	-	-	-
^10^	93	-	-	-
^12^	79.4	78.95	79.45	79.1
^13^	96	-	-	-
^9^	95.64	-	-	-
Proposed (DG-QFS)	96.73	96.87	96.70	96.67

Table 9 explains a comparative analysis of the proposed DG-QFS method with several existing approaches from the literature. The evaluation metrics, including Accuracy, Precision, Recall, and F1-Score, are measured. The proposed DG-QFS methodology outperforms some existing methods and scored the highest accuracy of 96.73%, along with superior Precision (96.87%), Recall (96.70%), and F1-Score (96.67%). In contrast, the earlier study^12^ reports an accuracy of 79.4%, but methods like^7,9^ achieve competitive performance with accuracies of 95.69% and 95.64%, respectively, but has accuracy lower than the DG-QFS model.

Nonetheless, the comprehensive performance of (DG-QFS) across all four metrics highlights its robustness and effectiveness in the context of Fetal US image classification.

The proposed (DG-QFS) framework significantly develops the fetal US plane classification by integrating multi-qubit entanglement score with dynamic graph-based feature selection. The features are extracted using a Mobile-Net architecture and refined through quantum circuits; the model achieves a high overall accuracy of 96.73%, along with consistently strong class-wise performance. Notably, the Brain and Cervix classes have attained perfect precision and recall (Brain: 99.86% precision, 97.96% recall; Cervix: 99.75% precision, 99.51% recall), which exhibits the model’s robustness in identifying the critical anatomical regions in the US. Therefore, the proposed DG-QFS model can be deployed in real-world clinical settings as a decision-support tool integrated into US imaging systems. After acquiring US images, the system extracts features using a lightweight Mobile-Net model and applies a quantum-enhanced feature selection, and classifies the fetal biometric plane in real time. This process helps the sonographers and clinicians to identify the key anatomical views in a fast manner and improves the diagnostic accuracy and efficiency. It can be implemented on cloud-based platforms or optimized edge devices for on-site use. With further validation, it can assist the standardized fetal assessments, especially in resource-limited or high-volume clinical environments.

## Current challenges and future directions

The proposed Dynamic Graph-Based Quantum Feature Selection (DG-QFS) framework demonstrates the performance with an overall accuracy of 96.73%. In Several aspects, it offers more advantages but has some limitations. The design reveals strong potential for extending its adaptability to diverse datasets and broader clinical scenarios. While the current implementation has been validated on a large, publicly available, and clinically annotated fetal ultrasound dataset, expanding the study to multi-center and multi-device data would further enhance generalization and robustness. The limitations observed in the “Femur” class are that it obtains 100% precision but has a lower recall (83.08%) by indicating a conservative prediction tendency. The “Other” class marked a high sensitivity (99.43% recall) and slightly lower precision (92.65%). It suggests an improvement in the specificity metric. Future work will concentrate on fine-tuning the quantum entanglement parameters to enhance performance in certain borderline classes and improve scalability on larger clinical datasets, thereby broadening applicability in real-time diagnostic settings.

## Conclusion

A novel Multi-level Quantum Entanglement-based Dynamic Graph Feature Selection (DG-QFS) design for US image classification, which integrates the strengths of quantum computing and graph-based learning. The DG-QFS method effectively identifies discriminative features through dynamic graph construction and quantum entanglement modeling and enhances the classification performance. Many experiments have demonstrated that DG-QFS outperforms the traditional feature selection techniques and baseline models across multiple evaluation metrics, achieving a maximum accuracy of 96.73%. The t-SNE visualization confirmed the superior separability of the selected features, and the ablation studies validated the effectiveness of each module present in the pipeline. Furthermore, comparisons with state-of-the-art methods in the literature survey establish the proposed method’s superiority in both accuracy and consistency. The computed results emphasize the potential of hybrid quantum-classical strategies in advancing medical image analysis and open avenues for future research in scalable and noise-resilient quantum models for clinical applications.

## Data Availability

The data and code supporting this study are available at: https://github.com/priyadharshni-code/(DG-QFS). The dataset used in this study is publicly available at 10.5281/zenodo.3904280. The dataset is openly accessible under the terms of the Creative Commons Attribution 4.0 International License.
